# Degradation of Atrazine in Water by Dielectric Barrier Discharge Combined with Periodate Oxidation: Enhanced Performance, Degradation Pathways, and Toxicity Assessment

**DOI:** 10.3390/toxics12100746

**Published:** 2024-10-14

**Authors:** Han Zhang, Jinping Duan, Pengcheng Luo, Luxiang Zhu, Yanan Liu

**Affiliations:** 1 College of Environmental Science and Engineering, Donghua University, 2999 North Renmin Road, Shanghai 201620, China; 1199121@mail.dhu.edu.cn (H.Z.); 15221771685@163.com (J.D.); 1229151@mail.dhu.edu.cn (P.L.); 2212061@mail.dhu.edu.cn (L.Z.); 2 Shanghai Institute of Pollution Control and Ecological Security, Shanghai 200092, China

**Keywords:** dielectric barrier discharge, periodate, atrazine, active species, decomposition pathway

## Abstract

The widespread occurrence of atrazine (ATZ) in water environments presents a considerable risk to human health and ecosystems. Herein, the performance of dielectric barrier discharge integrated with periodate (DBD/PI) for ATZ decomposition was evaluated. Results demonstrated that the DBD/PI system improved ATZ decomposition efficiency by 18.2–22.5% compared to the sole DBD system. After 10 min treatment, the decomposition efficiency attained 82.4% at a discharge power of 68 W, a PI dosage of 0.02 mM, and an initial ATZ concentration of 10 mg/L. As the PI dosage increased, the decomposition efficiency exhibited a trend of initially increasing, followed by a decrease. Acidic conditions were more favorable for ATZ removal compared to alkaline and neutral conditions. Electron paramagnetic resonance (EPR) was adopted for characterizing the active species produced in the DBD/PI system, and quenching experiments revealed their influence on ATZ decomposition following a sequence of ^1^O_2_ > O_2_^−^• > IO_3_• > OH•. The decomposition pathways were proposed based on the theoretical calculations and intermediate identification. Additionally, the toxic effects of ATZ and its intermediates were assessed. This study demonstrates that the DBD/PI treatment represents an effective strategy for the decomposition of ATZ in aquatic environments.

## 1. Introduction

Atrazine (ATZ) represents a typical triazine herbicide commonly employed in the control of grasses and broadleaf weeds in agriculture and forestry [1]. It is estimated that the worldwide usage of ATZ ranges from 70,000 to 90,000 tons annually [2]. ATZ has been detected in various aquatic environments owing to its high solubility and low biodegradability, with levels varying from ng/L to µg/L [2]. Several studies have reported that ATZ exhibits a range of toxic effects, including endocrine disruption, reproductive toxicity, and neurotoxicity, and is potentially carcinogenic to humans [3,4,5]. Previous research has demonstrated that conventional treatment technologies, including biodegradation, adsorption, nanofiltration, and coagulation, are either inefficient in eliminating ATZ or require additional disposal processes [6,7]. Thus, it is crucial to develop efficient methods for ATZ degradation from water.

Advanced oxidation processes (AOPs) are regarded as highly efficient methods for treating refractory wastewater, as they effectively decompose recalcitrant organic contaminants through the generation of highly reactive radicals. Typical AOPs used for ATZ removal include Fenton oxidation [8,9], photocatalytic oxidation [10], and ozonation [11,12]. However, Fenton-based oxidation typically requires a low pH range of 2~3.5 [13], restricting its practical application. The iron sludge produced during the treatment also needs additional disposal [10]. Solely ozone seems to be ineffective in removing ATZ and often requires integrating other processes to generate highly reactive OH• [11]. The photocatalytic oxidation process demonstrates efficient ATZ removal, yet many materials exhibit a strong preference for specific illumination [14]. Additionally, the stability and regeneration of the photocatalysts should also be considered. In recent years, plasma technology has become extensively used in treating wastewater, owing to its simple operational procedures, high efficacy in contaminant removal, and no secondary pollution. During the discharge process, high-energy electrons interact with gas or liquid molecules, resulting in the production of numerous active species like OH•, O_3_, H_2_O_2_, O_2_^−^•, and ONOO^−^ [15,16]. Nevertheless, the inadequate energy efficiency caused by the incomplete utilization of some active species, ultraviolet light, intense electric fields, and locally high temperatures generated during the discharge remains a considerable challenge in plasma wastewater treatment.

To improve these limitations, researchers have integrated plasma with oxidants or transition metals like hydrogen peroxide (H_2_O_2_) [17], ferrous ions (Fe^2+^) [18,19], persulfate (PS) [20,21], sulfite [19,22], and peracetic acid (PAA) [23,24] to further promote the production of active species. For instance, Shen et al. [20] utilized plasma for peroxydisulfate activation, significantly enhancing the ATZ removal efficiency, where SO_4_^−^• and OH• were confirmed as the functional active species responsible for ATZ decomposition. Likewise, Li et al. [23] employed the DBD/PAA system to degrade sulfamethoxazole in water, revealing that the energy yield of the DBD/PAA system was three times that of the DBD system alone. Periodate (PI) has recently gained considerable attention as an emerging oxidant due to its moderate oxidation potential, thermal stability, and convenient transport and storage [25,26]. Given the limited oxidative capacity of PI alone for effective contaminant decomposition, further activating PI to convert it into highly reactive free radicals, such as IO_3_•, OH•, and O_2_^−^•, can significantly improve its efficacy and facilitate the efficient decomposition of contaminants. So far, a variety of activation methods have been studied, including freezing [27], heating [28], alkali [29], hydrogen peroxide (H_2_O_2_) [30], ultraviolet (UV) radiation [31,32], ultrasound [33,34], and the use of transition metal ions and oxides [35,36]. Hence, the introduction of PI into the plasma system facilitates its activation through the application of various physical and chemical effects produced by plasma. This approach overcomes the weaknesses of sole plasma and sole PI treatment, thereby increasing the generation of a greater quantity of active species and enhancing the energy efficacy of the plasma process.

In this context, the performance of the DBD/PI treatment for ATZ decomposition was first studied. The effects of PI dosage, discharge power, initial ATZ concentration, and solution pH on ATZ removal were also investigated. Subsequently, electron paramagnetic resonance (EPR) and quenching experiments were conducted to identify the functional active species for ATZ decomposition. The intermediates of ATZ decomposition were analyzed by LC-MS, and potential decomposition pathways were suggested. Additionally, the toxicities of these intermediates were also predicted.

## 2. Materials and Methods

### 2.1. Chemicals

ATZ was provided by TCI (Shanghai, China). Sodium periodate (NaIO_4_), NaOH, HCl, tert-butanol (TBA), acetonitrile (HPLC grade), and methanol (HPLC grade) were provided by Sinopharm Chemical Reagent Co., Ltd. (Beijing, China). L-histidine and BQ were procured from Macklin Biochemical Co., Ltd. (Shanghai, China). Phenol, DMPO and TEMP were bought from Aladdin Biochemical Technology Co., Ltd. (Shanghai, China). All chemicals were of at least analytical purity and were utilized directly without additional purification.

### 2.2. Experimental Apparatus and Procedure

**Figure 1:**
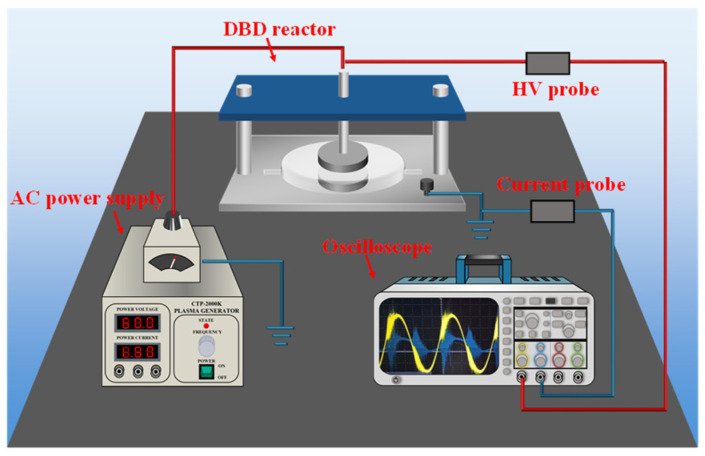
Schematic diagram of the experimental system.

**Figure 2:**
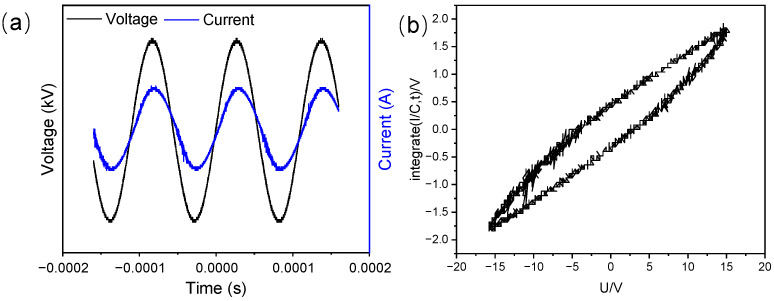
Discharge waveforms (**a**) and Lissajous figure (**b**) of the DBD/PI treatment.

Experiments were conducted utilizing the experimental apparatus depicted in Figure 1. The plasma generator (CTP-2000K) was provided by Nanjing Suman Plasma Technology Co., Ltd. (Nanjing, China). The DBD reactor was a planar quartz glass container, 150 mm in diameter and 8 mm in thickness. The reactor was connected to a high-voltage electrode at the top and a grounding electrode at the bottom. An oscilloscope (TBS2104B, Tektronix, Beaverton, OR, USA) was used to monitor the discharge parameters, and the discharge waveforms were illustrated in Figure 2.

ATZ stock solution of 1 g/L was prepared in acetonitrile and stored at 4 °C before use. During the experiment, the stock solution was diluted with deionized water until the desired concentration (5 mg/L to 25 mg/L) was reached. In each experimental batch, 35 mL of ATZ solution was introduced into the reactor for treatment of 2, 4, 6, 8, and 10 min. following the treatment, the samples were subjected to filtration utilizing a 0.45 µm polytetrafluoroethylene membrane for subsequent analysis. Each experiment was performed at least in duplicate, and the average data were presented.

### 2.3. Analytical Methods

ATZ was quantified using a UltiMate 3000 (Thermo Fisher, Waltham, MA, USA) high-performance liquid chromatography (HPLC) equipped with an ODS C18 column (4.6 × 250 mm). The mobile phase was methanol and ultrapure water (60/40, *v*/*v*) and the flow rate was fixed at 1 mL/min. The injected sample volume was 20 µL. The detection wavelength was 225 nm, and the temperature of analytical column was maintained at 35 °C.

The intermediates of ATZ decomposition were analyzed by LC-MS (Agilent 1290 UPLC-QTOF 6550, Agilent, CA, USA) equipped with a Waters BEH C18 column (2.1 × 100 mm). Formic acid water (0.1%) (A) and acetonitrile (B) served as the mobile phase. The elution procedure began with 90% mobile phase A and held for 10 min, then decreasing linearly to 10% in 20 min, followed by an immediate rise to 90% in 1 min and sustained for 4 min. A total of 5 µL of sample was injected for analysis and the column temperature was 35 °C. QTOF system operated in positive ion mode electrospray ionization (ESI) mode with a voltage of 4000 V and scanning range of 50~1000 *m*/*z*. The sheath gas temperature was 350 °C, and the gas flow rate was 12 L/min.

The decomposition kinetics were determined based on the pseudo-first-order kinetic model using Equation (1):

(1) $$\ln\frac{C_{t}}{C_{0}}=-\mathrm{kt}$$

where C_0_ and C_t_ were the concentrations of ATZ at 0 min and t min, mg/L; k was the pseudo-first-order rate constant, min^−1^.

The synergistic impact between DBD and PI was confirmed by calculating the synergistic factor (SF) through Equation (2):

(2) $$\mathrm{SF}=\frac{k_{\mathrm{DBD}/\mathrm{PI}}}{k_{\mathrm{DBD}\;}+k_{\mathrm{PI}}}$$

where k_DBD/PI_ was the rate constant of ATZ decomposition in the DBD/PI system; k_DBD_ and k_PI_ were the rate constants of ATZ decomposition in the sole DBD and PI systems, respectively.

Discharge power was calculated using Equation (3) according to Lissajous figure:

(3) $$P=\frac{1}{T}\int_{0}^{T}\mathrm{VI}\mathrm{dt}=\;\frac{C_{M}}{T}\int_{0}^{T}V\frac{dV_{M}}{\mathrm{dt}}\mathrm{dt}=fC_{M}\int \mathrm{Vd}V_{M}$$

where P was the discharge power, W; V was the output voltage of the power supply, V; T was the period; f was the output frequency, Hz. When V and V_M_ were plotted on the *x*-*y* axes of the oscilloscope, a closed curve, known as the Q-V Lissajous figure, is obtained as shown in Figure 2b. The area of this closed curve is directly proportional to the discharge power, P.

Assume that the sensitivity of the *X*-axis of the oscilloscope is kx volts per grid, the sensitivity of the *Y*-axis is ky volts per grid, and the ratio of the voltage divider is k. Then the area enclosed by the Lissajous figure is A. From Equation (3), the discharge power is:

(4) $$P=fC_{M}\mathrm{kxkykA}$$

The internal sampling capacitor of the experimental power supply is C = 0.47 µF, and the voltage sampling ratio is 1000:1, meaning the actual voltage unit is in kilovolts (kV), with k = 1000. The signal acquisition attenuation factors for CH1 and CH2 are kx = 1 and ky = 1 (This experiment has been calibrated and there is no attenuation). The Lissajous figure area, A = 17.1, was directly calculated using Origin 2024 software (OriginLab, Northampton, MA, USA).

Energy yield (EY, mg/kWh) refers to the quantity of ATZ degraded by consuming one unit of energy and was determined using Equation (5) [37]:

(5) $$\mathrm{EY}=\frac{C_{0}\;\times \;V\;\times \;\eta }{P\;\times \;t}$$

where C_0_ was the ATZ concentration (mg/L), η was the ATZ removal efficiency (%), P is the discharge power (W), V was the volume of ATZ solution (mL), and t is the discharge time (h), respectively.

DFT calculations were carried out utilizing the Gaussian 16 Revision C.02 program [38]. ATZ molecular geometry optimization and vibrational frequency analysis were run at the M06-2X/def2-SVP level [39]; single-point energy calculations were run at M06-2X/def2-TZVP level employing the SMD model.

The solution pH was determined utilizing a pH meter (6010M, Jenco). The existence of free radicals was identified using EPR spectrometer (EMXnano231, Bruker, Germany). The toxicity assessment of the intermediates was performed using ECOSAR v2.2 toxicity prediction software [40].

## 3. Results and Discussion

### 3.1. Decomposition of ATZ by DBD Combined with PI

**Figure 3:**
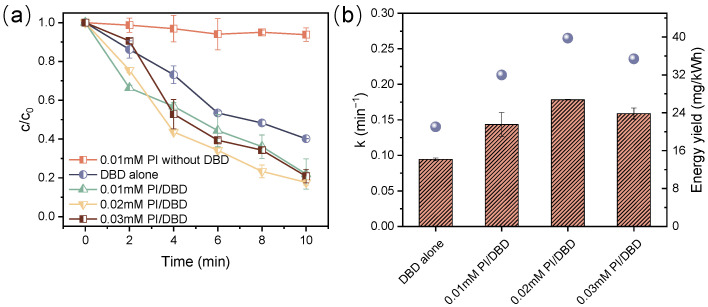
Relative concentration decay curve for ATZ decomposition (**a**); the pseudo-first-order rate constants and energy yields at different PI dosages (**b**) ([ATZ] = 10 mg/L, P = 68 W).

Figure 3 illustrates the decomposition of ATZ through plasma treatment with and without the addition of PI. In the control experiment with PI alone, the decomposition efficiency of ATZ was merely 6.2%, suggesting that PI itself exerted a very limited oxidative capacity for ATZ decomposition. Following a 10-min of sole DBD treatment, the concentration of ATZ in the solution decreased by approximately 59.8%, while this value increased to 78.0%, 82.4%, and 79.2% upon the introduction of 0.01, 0.02, and 0.03 mM PI. Correspondingly, the rate constant (k) of ATZ decomposition rose from 0.094 to 0.14, 0.18, and 0.16 min^−1^, respectively. The calculated synergistic factor (SF) for the DBD treatment and PI with different concentrations ranged from 1.41 to 1.77, suggesting that the DBD/PI system exhibited a good synergistic impact on pollutant removal. Furthermore, the energy yields of the sole DBD and DBD/PI system were determined, as illustrated in Figure 3b. The energy yield of the DBD/PI systems (32.0~39.8 mg/kWh) was 1.52~1.89 times that of the sole DBD system (21.0 mg/kWh), demonstrating that the integration of PI enhanced the energy efficiency of the plasma process. The observed results could be elucidated by the activation of PI by plasma, which promoted the production of active species and accelerated ATZ decomposition. Specifically, ultraviolet light and heat generated during the plasma process enabled PI to be effectively activated to form IO_3_• and O^−^• (Equation (6)) [25]. Furthermore, PI could also directly interact with the active species produced in the plasma system. For instance, H_2_O_2_ can react with IO_4_^−^ to yield IO_3_• and O_2_^−^•, followed by the radical rearrangement and recombination reactions, ultimately leading to the formation of OH• and ^1^O_2_ (Equations (7)–(10)) [41,42].

IO_4_^−^ + hv/heat → IO_3_• + O^−^• (6)

IO_4_^−^+ H_2_O_2_ → IO_3_• + O_2_^−^• + H_2_O (7)

IO_4_^−^ + 2O_2_^−^• + H_2_O → IO_3_^−^ + 2^1^O_2_ + 2OH^−^ (8)

H_2_O_2_ + O_2_^−^• → OH• + OH^−^ + ^1^O_2_ (9)

IO_3_^−^ + •OH → IO_3_• + OH^−^ (10)

Notably, while the introduction of an appropriate amount of PI promoted the generation of active species in the DBD system, an excessive concentration of PI addition may exert detrimental effects on the decomposition process. Figure 3 illustrates a slight decrease in ATZ decomposition as the additive PI dose escalated from 0.02 to 0.03 mM, with the decomposition efficiency and k value decreasing by 3.2% and 11.0%, respectively. Excess IO_4_^−^ not only quenched the OH• generated in the system but also reacted with IO_3_• (Equations (11) and (12)), thus competing with ATZ for the active species in the system. Moreover, the recombination of iodine radicals also reduced the abundance of active species (Equations (13) and (14)) [43], thus limiting the decomposition of ATZ.

IO_4_^−^ + •OH → IO_4_• + OH^−^ (11)

IO_4_^−^ + IO_3_• → IO_3_^−^ + IO_4_• (12)

IO_3_• + IO_3_• → I_2_O_6_ (13)

IO_4_• + IO_4_• → I_2_O_8_ (14)

### 3.2. Effect of Discharge Power

**Figure 4:**
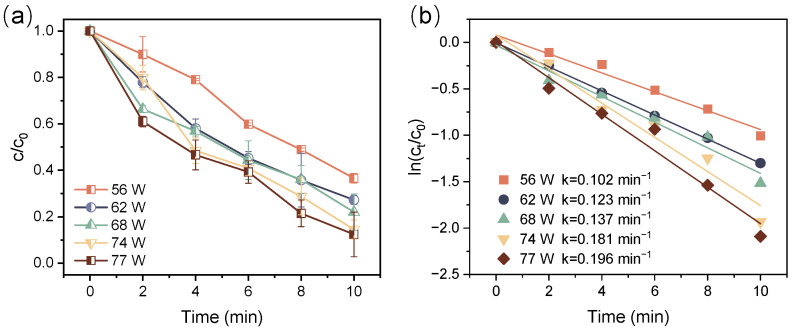
Relative concentration decay curve for ATZ decomposition (**a**) and kinetic curves (**b**) at different discharge powers ([ATZ] = 10 mg/L, [IO_4_^−^] = 0.01 mM).

The discharge power greatly influences the abundance of active species and the intensity of physical effects, which are crucial for the decomposition of contaminants [44]. Figure 4 presents the ATZ decomposition efficiency and the corresponding k values in the DBD/PI system at different discharge powers. At a discharge power of 56 W, the removal efficiency of ATZ was 63.4% after 10-min treatment, while it enhanced to 72.8%, 78.0%, 85.6%, and 87.6% at discharge power of 62, 68, 74, and 77 W, respectively. Correspondingly, the k values rose from 0.102 to 0.196 min^−1^ as the discharge power escalated from 56 to 77 W. The enhancement of electric field strength with greater discharge power could lead to increased electron density and greater amounts of active species, thus increasing the likelihood of collisions between ATZ and electrons or active species [45]. More importantly, the enhanced physical and chemical effects produced at higher discharge power further facilitated the activation of PI, resulting in the generation of more active species that promoted the decomposition of ATZ.

### 3.3. Effect of Initial ATZ Concentration

**Figure 5:**
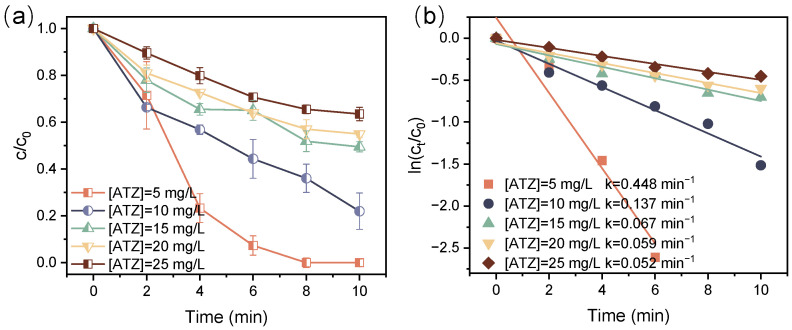
Relative concentration decay curve for ATZ decomposition (**a**) and kinetic curves (**b**) at different initial ATZ concentrations ([IO_4_^−^] = 0.01 mM, P = 68 W).

Figure 5 demonstrates the decomposition efficiency of ATZ at various initial concentrations in the DBD/PI system. The decomposition efficiency and k value of ATZ were observed to decrease with increasing initial concentration. As shown in Figure 5a, the decomposition efficiency of ATZ achieved 100% within 8-min treatment at an initial concentration of 5 mg/L, yet it fell to 78.0%, 50.5%, 45.0%, and 36.5% as the initial concentration increased to 10, 15, 20 and 25 mg/L, respectively. At a certain discharge power, the quantity of active species generated in the DBD/PI system remained steady [46]; thus, an increase in ATZ initial concentration resulted in heightened competition for active species. This increased competition ultimately led to insufficient active species within the system to effectively degrade the excess ATZ. Additionally, the intermediates produced during the decomposition process would further compete with ATZ for active species. Nevertheless, the absolute removal amount at an initial concentration of 25 mg/L was twice that of 5 mg/L, which could be ascribed to the increased collision probability between active species and ATZ at higher initial concentrations, thus improving the utilization efficacy of active species.

### 3.4. Effect of Solution pH

**Figure 6:**
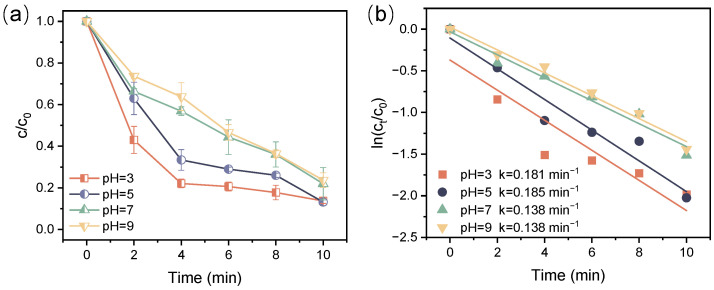
Relative concentration decay curve for ATZ decomposition (**a**) and kinetic curves (**b**) at different solution pH ([ATZ] = 10 mg/L, [IO_4_^−^] = 0.01 mM, P = 68 W).

Solution pH is a vital factor influencing the contaminant decomposition in the plasma process, as it impacts both the formation of active species and the protonation of the contaminants. In the presence of PI, solution pH also affects PI speciation and oxidation potential. As illustrated in Figure 6, the decomposition efficiencies of ATZ in the DBD/PI system were 86.3%, 86.8%, 78.0%, and 76.3% at pH 3, 5, 7 and 9, respectively. Correspondingly, the k values declined from 0.181 to 0.138 min^−1^ when the solution pH rose from 3 to 9. In general, ATZ achieved effective decomposition across the whole pH range, with acidic conditions being more favorable for its decomposition. Given that the pKa value of ATZ was approximately 1.7, it maintained negative charges across the pH range of 3~9 [47]. Thus, the influence of solution pH was likely due to its impact on the active species and PI species formation in the system. On the one hand, a greater quantity of OH• was generated within the system under acidic conditions, and it maintained a higher oxidation potential in this case [48,49], thereby promoting the decomposition of ATZ. In contrast, OH• was readily consumed by excess OH^−^ in alkaline solutions. On the other hand, the dominant species of PI varied with the pH of the solution. Specifically, the dominant species of PI was IO_4_^−^ when the solution pH < 8, while it was converted to the dimerized H_2_I_2_O_10_^4−^ when the solution pH exceeded 8 [50]. This variation in species also led to a reduction in PI oxidation potential from 1.6 eV to 0.7 eV [43], thereby hindering the decomposition of ATZ.

### 3.5. The Roles of Active Species

**Figure 7:**
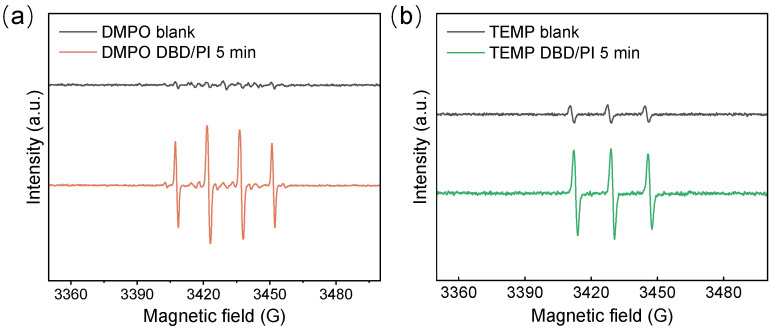
EPR spectra of (**a**) DMPO and (**b**) TEMP as spin-trapping agents in the DBD/PI system.

The active species formed in the system were verified using EPR. Figure 7a illustrates that adding DMPO to the DBD/PI system produced a signal with a distinct 1:2:2:1 characteristic peak, signifying the generation of OH• within the system. OH• is the major reactive radical in the DBD system, possessing an oxidation potential of 2.8 V, and is likely to be significantly involved in the decomposition of ATZ [51]. Notably, while DMPO can also capture O_2_^−^•, the rapid decomposition of DMPO-OOH into DMPO-OH presents significant challenges for its detection. Moreover, when TEMP was added to the solution, a strong 1:1:1 characteristic signal was detected, demonstrating the presence of ^1^O_2_ in the DBD/PI system (Figure 7b).

**Figure 8:**
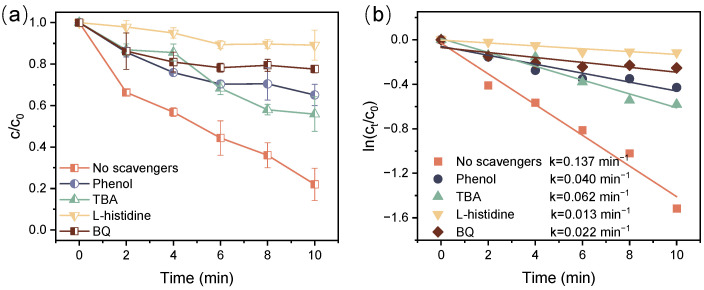
Relative concentration decay curve for ATZ decomposition (**a**) and kinetic curves (**b**) with different scavengers addition ([ATZ] = 10 mg/L, [IO_4_^−^] = 0.01 mM, P = 68 W).

To further investigate the functional active species of ATZ decomposition in the DBD/PI system, TBA, L-histidine, and BQ were employed to scavenge OH•, ^1^O_2_, and O_2_^−^•, respectively. As displayed in Figure 8, the ATZ decomposition efficiency dramatically dropped from 78.0% to 10.9% with the addition of 30 mM L-histidine, and the corresponding k value decreased by 90.7%, suggesting that ^1^O_2_ exhibited an essential contribution to ATZ decomposition. It was reported that ^1^O_2_ possesses high electrophilicity, allowing it to selectively react with electron-rich compounds [52]. This enables it to interact with the N-isopropyl group in the ATZ molecule, thereby promoting the decomposition of ATZ [53]. The decomposition of ATZ was also strongly inhibited when TBA and BQ were added to the DBD/PI system, and the decomposition efficiencies decreased from 78.0% to 44.1% and 22.4%, respectively, suggesting that OH• and O_2_^−^• were also crucial in ATZ decomposition in the DBD/PI system. Moreover, the quenching of O_2_^−^• and OH• may also influence the activation of PI [25], consequently leading to a further reduction in ATZ decomposition. Additionally, the formation of iodine radicals through the activation of PI is expected, yet direct identification remains challenging. Thus, to explore the impact of IO_3_• on the ATZ decomposition, the quenching experiment was also conducted using phenol as a radical scavenger [42]. Figure 8 demonstrates that phenol addition significantly hindered the decomposition of ATZ, resulting in a reduction of decomposition efficiency from 78.0% to 34.9%. This observation suggested that IO_3_• was also responsible for the decomposition of ATZ. In general, these scavengers exhibited inhibitory impacts following the order of L-histidine > BQ > phenol > TBA, suggesting that the contributions of active species to ATZ decomposition during the DBD/PI treatment were ranked in the following order: ^1^O_2_ > O_2_^−^• > IO_3_• > OH•.

### 3.6. Degradation Mechanism of ATZ

**Figure 9:**
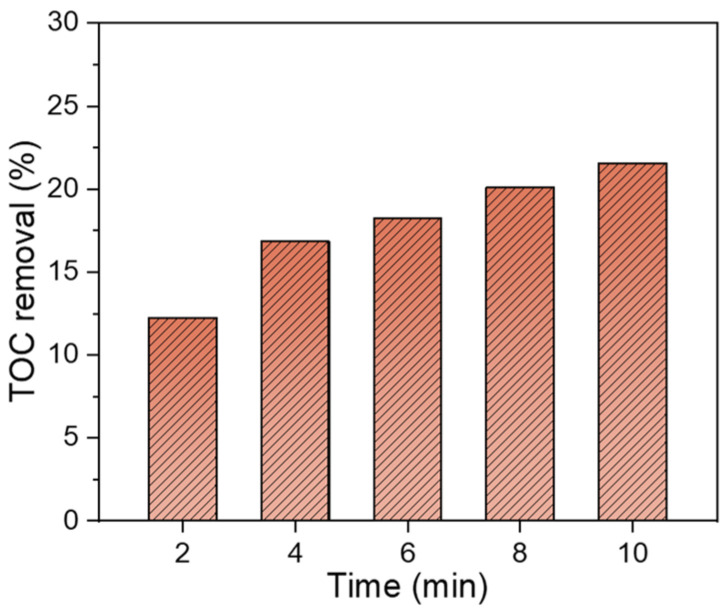
TOC removal during ATZ degradation in the DBD/PI system (pH = 7.0, [ATZ] = 10 mg/L, [IO_4_^−^] = 0.01 mM, P = 68 W).

Figure 9 illustrates the ATZ mineralization during the DBD/PI process. The total organic carbon (TOC) removal increased with discharge time, reaching approximately 21.5% after 10 min treatment. This result suggested that some organic intermediates were formed during the ATZ degradation process.

**Figure 10:**
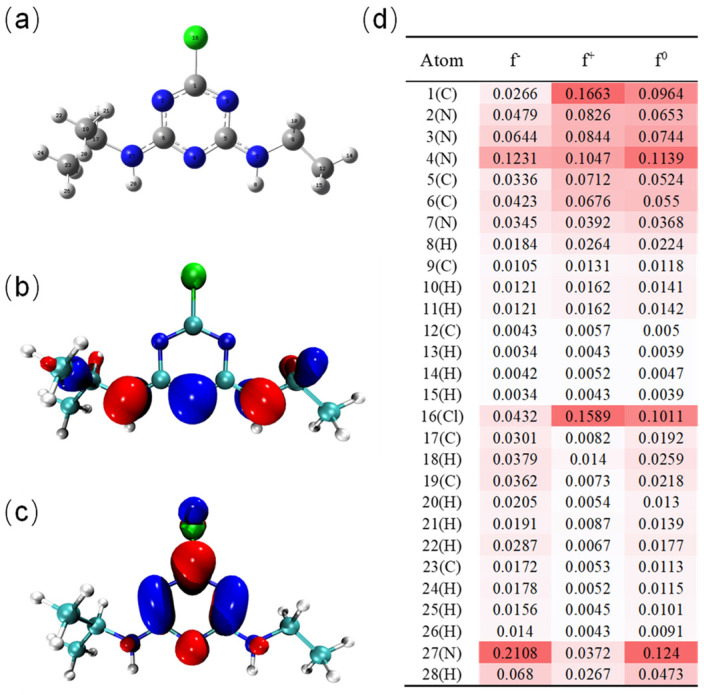
The optimized chemical structure of ATZ (**a**), HOMO (**b**), LUMO (**c**) and Fukui index (f^−^, f^+^, and f^0^ represent electrophilic, nucleophilic, and radical attacks) (**d**).

Therefore, density functional theory (DFT) calculation combined with intermediate identification was conducted to further explore the degradation mechanism of ATZ. The highest occupied and lowest unoccupied molecular orbital (HOMO and LUMO), as well as the Fukui function of ATZ, were first calculated. Figure 10b,c illustrates that the HOMO of ATZ was mainly distributed on the C-N, C-H, and C-C bonds of the side chain, whereas the LUMO was concentrated on the triazine ring and the C-Cl bond, suggesting that they were susceptible to electrophilic and nucleophilic reactions, respectively. Figure 10d presents the detailed Fukui indices of ATZ. The result revealed that N27, N4, and H28 exhibited higher f^−^ values, implying their susceptibility to electrophilic attack; C1, Cl16, and N4 showed higher f^+^ values, suggesting that they were prone to nucleophilic attack. Likewise, N27, N4, Cl16, and C1 demonstrated the highest f^0^ values, signifying that they were also prone to react with radicals.

Subsequently, the degradation intermediates were analyzed via liquid LC-MS and a total of nine intermediates were identified. Based on theoretical calculations and the identified intermediates, the possible decomposition pathways of ATZ were proposed. Firstly, the electrophilic character of ^1^O_2_ and IO_3_• enables them to target electron-rich functional groups in contaminants [54,55]. Thus, the N atoms present in the side chain of ATZ were readily susceptible to attack by ^1^O_2_/IO_3_•, leading to the cleavage of the C-N bond. On the other hand, IO_3_• and OH• transformed ATZ into the carbon-centered radical through hydrogen atom abstraction, which subsequently interacted with O_2_/O_2_^−^• to yield peroxide radical [6,56]. These processes ultimately led to the formation of 6-chloro-N^2^-ethyl-N^4^-methyl-1,3,5-triazine-2,4-diamine (CEAT) and 6-chloro-N^2^-isopropyl-1,3,5-triazine-2,4-diamine (CAIT) via dealkylation. The oxygen-attacked carbon-centered radical could also be dehydrated to form the olefination by-products 6-chloro-N^2^-isopropyl-N^4^-vinyl-1,3,5-triazine-2,4-diamine (CVIT) and 4-isopropylamino-6-vinylamino-1,3,5-triazin-2-ol (OVIT) [52]. CEAT further underwent alkylic-oxidation to form 2-chloro-4-amino-6-acetamido-s-triazine (CADT). CAIT and OVIT could be further dealkylated to yield 2-chloro-4,6-diamino-s-triazine (CAAT) and 2-chloro-4-amino-6-acetamido-s-triazine (OAIT), respectively [47]. In addition, the Cl atom in the ATZ molecule was susceptible to attack by plasma electrons or OH• due to its high f^0^ and f^+^ values, leading to the formation of 4-ethylamino-6-isopropylamino-s-triazine (OEIT), while 2-hydroxy-4,6-diamino-s-triazine (OAAT) was also generated as the reaction progressed.

**Figure 11:**
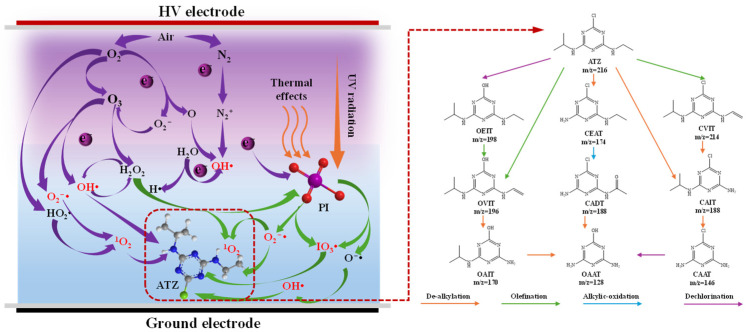
The proposed mechanism diagram of ATZ decomposition in the DBD/PI system.

Based on the aforementioned discussion, we proposed a possible mechanism for PI addition to improve the plasma degradation of ATZ, as shown in Figure 11. High-energy electrons in plasma dissociated or excited gas molecules to form excited species and radicals, which subsequently initiate a series of chain reactions to generate various active species when in contact with water. Introducing PI into the system further promoted the generation of active species. PI was activated by plasma electrons, H_2_O_2_, UV radiation, and thermal effects produced by plasma discharge, causing the generation of IO_3_•. Subsequently, the generation of OH•, O_2_^−^•, and ^1^O_2_ was intensified through propagation reactions. Finally, under the attack of these active species, ATZ was degraded via de-alkylation, olefination, alkylic-oxidation, and dechlorination reactions.

### 3.7. Toxicity Analysis

**Figure 12:**
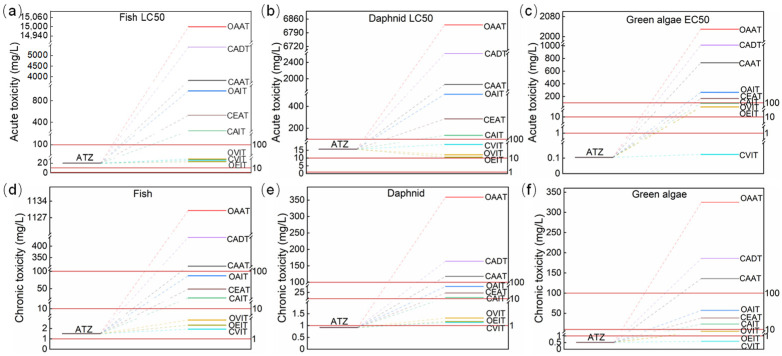
Predicted acute toxicities (**a**–**c**) and chronic toxicities (**d**–**f**) for fish, daphnid and green algae of ATZ and its intermediates by ECOSAR.

To explore the ecological impact of ATZ and its intermediates, the acute and chronic toxicity of ATZ and its intermediates were predicted by using ECOSAR v2.2 [40], as shown in Figure 12.

In terms of acute toxicity, the LC_50_ values of ATZ were 20.2 mg/L for fish and 15.7 mg/L for daphnid, while the EC_50_ value for green algae was 0.105 mg/L. For chronic toxicity, the chronic values (Chv) of ATZ were 1.5, 0.917, and 0.51 mg/L for fish, daphnids, and green algae, respectively. These results suggested that ATZ can be categorized as harmful to fish, toxic to daphnids, and very toxic to green algae. Following the treatment of the DBD/PI system, reduced acute toxicity in seven intermediates compared to ATZ was observed. Additionally, the chronic toxicity of all intermediates was lowered in contrast to ATZ, suggesting that the DBD/PI treatment effectively mitigated the adverse impacts of ATZ on aquatic ecosystems.

## 4. Conclusions

The integration of DBD and PI enhances the decomposition of ATZ and increases the energy efficacy of the plasma treatment process. The influence of PI dosage, discharge power, initial ATZ concentration, and solution pH value on the decomposition process was studied. Increased discharge power and reduced initial concentration were favorable for ATZ decomposition. The decomposition efficiency demonstrated an initial upward trend followed by a decline as the PI concentration increased, with the best performance observed at a PI concentration of 0.02 mM. Acidic environments were more favorable for ATZ decomposition by the DBD/PI system compared to alkaline and neutral conditions. EPR analysis and quenching experiments confirmed that ^1^O_2_, OH•, O_2_^−^•, and IO_3_• significantly contribute to the decomposition of ATZ. Based on the reactive sites analysis and intermediate identification results, the decomposition pathways were proposed involving de-alkylation, olefination, alkylic-oxidation, and dechlorination. Additionally, the toxicities of the intermediates were evaluated. This study reveals that the DBD/PI process is a viable approach for treating ATZ-contaminated wastewater, and it offers insights for enhancing plasma-based treatment in wastewater remediation.

## Data Availability

Data will be made available on request.
